# Prevention of acute radiation-induced Proctitis by *Aloe vera*: a prospective randomized, double-blind, placebo controlled clinical trial in Pelvic Cancer patients

**DOI:** 10.1186/s12906-020-02935-2

**Published:** 2020-05-13

**Authors:** Adeleh Sahebnasagh, Arash Ghasemi, Jafar Akbari, Abbas Alipour, Hossein Lashkardoost, Shahram Ala, Seyed Jalal Hosseinimehr, Ebrahim Salehifar

**Affiliations:** 1 Clinical Research Center, Department of Internal Medicine, Faculty of Medicine, North Khorasan University of Medical Sciences, Bojnurd, Iran; 2 Emam Khomeini Hospital, Mazandaran University of Medical Sciences, Sari, Iran; 3 Pharmaceutical Research Center, Faculty of Pharmacy, Mazandaran University of Medical Sciences, Sari, Iran; 4 Epidemiology, Faculty of Medicine, Community medicine Department, Mazandaran University of Medical Sciences, Sari, Iran; 5 School of Public Health, North Khorasan University of Medical Sciences, Bojnurd, I.R Iran; 6 Department of Radiopharmacy, Faculty of Pharmacy, Mazandaran University of Medical Sciences, Sari, Iran; 7 Gastrointestinal Cancer Research Center, Faculty of Pharmacy, Mazandaran University of Medical Sciences, Sari, Iran; 8 Department of Clinical Pharmacy, Faculty of Pharmacy, Mazandaran University of Medical Sciences, 48471-16548, Km 18 Khazarabad Road, Khazar sq., Sari, Mazandaran Province Iran

**Keywords:** Acute radiation Proctitis, *Aloe vera*, Complications, Pelvic neoplasms, Prevention

## Abstract

**Background:**

Acute radiation-induced proctitis (ARP) is the most common side effect following radiotherapy for malignant pelvic disease. This study evaluated the efficacy of *Aloe vera* ointment in prevention of ARP.

**Methods:**

Forty-two patients receiving external-beam radiotherapy (RT) for pelvic malignancies were randomized to receive either *Aloe vera* 3% or placebo topical ointment during radiotherapy for 6 weeks. These patients were evaluated based on the severity (grade 0–4) of the following symptoms weekly: rectal bleeding, abdominal/rectal pain, diarrhea, or fecal urgency. RTOG acute toxicity criteria and psychosocial status of the patients were also recorded weekly. Lifestyle impact of the symptoms, and quantitative measurement of C-reactive protein (CRP), an indicator of systemic inflammation, were also measured.

**Results:**

The results of present study demonstrated a significant preventive effect for *Aloe vera* in occurrence of symptom index for diarrhea (*p* < 0.001), rectal bleeding (p < 0.001), and fecal urgency (*p* = 0.001). The median lifestyle score improved significantly with *Aloe vera* during RT (p < 0.001). Intervention patients had a significant lower burden of systemic inflammation as the values for quantitative CRP decreased significantly over 6 weeks of follow-up (*p* = 0.009).

**Conclusion:**

This study showed that *Aloe vera* topical ointment was effective in prevention of symptoms of ARP in patients undergoing RT for pelvic cancers.

**Trial registration:**

IRCT201606042027N6. Registration date: 2016-09-04.

## Background

Acute radiation-induced proctitis (ARP) is the most common side effect following radiotherapy (RT) for malignant pelvic diseases. This injury may occur in ≤75% of patients [1, 2]., The symptoms are including diarrhea, rectal bleeding and pain, abdominal cramping, mucoid discharge, and fecal urgency. The acute symptoms usually resolve after cessation of RT within a few months, but a small number of patients experience chronic injury, which has an additional negative influence on patients’ daily functions and quality of life [3–7],. ,A,RP may take place during treatment or as soon as RT ends. It is the result of direct cellular damage, causing loss of cellular function and mucosal inflammation. Chronic radiation proctitis, however, appears about months to years after the initial radiation exposure. The interaction of cells with radiation may cause an enhanced production of reactive oxygen species (ROS) and radical products. Therefore, the molecular mechanism of this injury is thought to be due to free oxygen metabolites [8]. A variety of prophylactic and therapeutic modalities have been proposed for the management of radiation-induced proctitis, but there is no widely accepted prophylactic or effective treatment for proctitis [9].

*Aloe vera* has been used for centuries for its health, beauty, medicinal and skin care properties. It contains 75 potentially active constituents, including polysaccharides, anthraquinone, lectin, superoxide dismutase, glycoprotein, vitamins C and E, salicylic acids and amino acids [10]. Traditionally, *Aloe vera* has been used topically in cosmetic products and herbal remedies in treatment of a range of inflammatory skin diseases for its anti-inflammatory, analgesic, wound healing, scavenging free radicals, antiproliferative, anticarcinogenic and antiaging properties [11, 12]. It is believed that *Aloe vera* exerts its anti-inflammatory effects through suppression of cyclooxygenase-2. It has also been used successfully for thermal burns, traumatic surgical wounds, radiation-induced dermatitis and skin ulceration [13]. In addition, this medicinal herb has been successfully used in treatment and alleviating ARP symptoms in patients receiving RT. [14] *Aloe vera* mouthwash has shown promised efficacy in prevention and alleviation of radiation-induced mucositits in head and neck cancers without marked side effects [15, 16]. In other clinical studies, topical *Aloe vera* was compared to silver sulfadiazine or Vaseline gauze in treating burn wound. The average time of healing reduced significantly in Aloe group [17, 18]. *Aloe vera* cream has been also applied post-hemorrhoidectomy for ameliorating pain and recovering epithelial in order to accelerate wound healing [19]. Seborrheic dermatitis is an inflammatory skin disorder with high prevalence. *Aloe vera* extract has been shown to be effective in this dermal condition, too [20]. *Aloe* extracts has proven antioxidant activity which protects against ROS-induced membrane and cellular damage [21]. In previous clinical studies, a significant number of patients with chronic radiation proctitis seemed to benefit from combination anti-oxidant therapy of Vitamin C and E with sustained improvement in their clinical symptoms for as long as 1 year [8, 22]. Although the mechanism of action by which *Aloe vera* might facilitate healing is not clearly delineated, one hypothesis is that it exerts its effects as a result of antioxidant and immunomodulatory properties, and cyclooxygenase-2 suppression [23]. One of active compounds in Aloe is salicylic acid, which can be converted into salicylate and thereby inhibits prostaglandin synthesis and the resulting inflammation [24]. Therefore, because of mentioned pathophysiology of radiation-induced proctitis and potential anti-inflammatory and anti-oxidant properties of *Aloe vera*, we hypothesized that it may be beneficial in prevention of radiation proctitis.

This is the first randomized study of *Aloe vera* in prevention of radiation proctitis in patients undergoing RT to the pelvis. Using this trial, we examined whether *Aloe vera* could significantly prevent the incidence of radiation-induced proctitis, compared with placebo. In addition to our primary end point, development of proctitis, we evaluated secondary end points of quality of life (QOL), psychosocial status using Hospital Anxiety-Depression Scale (HAD), and also quantitative measurement of C - reactive protein (CRP).

## Materials and methods

This clinical trial was registered by Iranian Registry of Clinical Trials (IRCT201606042027N6). This was a randomized, double-blind, prospective, placebo-controlled trial comparing the preventive effects of *Aloe* ointment with placebo ointment. It was carried out in Department of Radiation Oncology (Imam Hospital, Sari, Iran). This study adheres to CONSORT guidelines. The inclusion criteria for enrollment were as follow: all patients aging 18 years or more, who were undergoing radiation for pelvic malignancies.

Exclusion criteria were evidence of active infection; evidence of other sources of hematochezia including colon cancer, inflammatory bowel disease, and hemorrhoids; anal incontinence, anorectal fistula, anorectal stenosis; previous rectal surgery; pregnancy or breast feeding, female of child-bearing age not taking adequate contraception; known allergy to any ingredients of the ointment; and concomitant use of antibiotics or steroids.

### *Aloe vera*

We applied pure spray-dried *Aloe vera* powder (Giah salamat nasim faraz, Fars, Iran). This product consists of the inner gel from plants. Vaseline and liquid white paraffin were purchased from Sina Tolid (Tehran, Iran).

### Preparation and formulation of *Aloe vera* ointment

At baseline, we tested *Aloe vera* powder for any microbial contaminants. Since the culture results were positive and also the product was spray-dried, gamma waves were applied for sterilization of the powder (by Atomic Energy Organization of Iran).

In order to formulate the ointment, first, all the containers and equipment were sanitized. The baseline step was grinding 9 g *Aloe vera* with a mortar to yield a very fine powder. After levigating with 7 g liquid white paraffin, the mixture was blended with 284 g Vaseline by geometric method and *Aloe vera* 3% ointment was prepared. To obtain smooth and super fine blending ointment, the whole mass was passed through mill (triple roller ointment mill ointment grinding machine, D-63150; Erweka, Heusenstamm, Germany). In the end, the tubes were filled under aseptic laminar flow hood. Each tube weighed 50 g. Placebo was prepared using the same method for *Aloe* ointment; but it was vehicle only. Excipients and appearance of the placebo ointment were the same.

### Study design

In this prospective study, double-blind randomization was used. Patients were assigned to receive either *Aloe vera* or placebo ointment. Participants applied the ointments rectally via applicator, from the first day of starting radiotherapy and for 6 weeks, 1 g twice daily.

The ointment lacquered aluminum collapsible tubes looked identical. Each ointment was given a six-digit random number by the principal investigator. Patients, clinical oncologist and investigator of clinical responses were all blind to the arms of the study. At the end of the study, the principal investigator decoded the numbers of consumed ointments and assigned each to the appropriate group correctly. Patients were allocated to the study group according to Random Number Table. Patients were assessed on a weekly basis during radiotherapy. Compliance was assessed by asking the patients to bring the rest of their tubes for weekly visits. For management of missing data, if we could record 90% of the clinical responses in patients during the 6-week follow-up, we included them in the study and intension-to-treat analysis was done. Otherwise, the patients were excluded. Questionnaires on demographic and clinical characteristics of each patient were obtained by a single investigator. It included age, sex, cancer diagnosis, co-morbidity, previous treatments and the radiation dose.

### Primary endpoint

The major outcome criteria of the study were prevention of ARP development, based on clinical symptoms investigated on weekly evaluation. Radiation-induced toxicity was evaluated weekly using RTOG criteria and based on clinical presentation [14]. Four signs of ARP were recorded as follow: rectal/abdominal pain, rectal bleeding, stool inconsistency and fecal urgency. For each factor, a scale from 0 (not present) to 4 (causing significant discomfort) was used. The maximum overall score was 16. We considered the severity of the sum of these four components as a series of prevention effects of our interventions.

### Radiation treatment

External beam radiotherapy was given as single daily doses of 1.8–2 Gy to a total dose of 41–72 Gy over 6.5–7 weeks, given 5 days per week. Individual computed tomography-based planning was applied in our department. Customized blocks were used for rectal shielding. Patients were treated in supine position. Prostate tumors penetrating the capsule (T3, T4), tumors of the urinary bladder and uterine cervical cancers were treated with pelvic fields to 45 Gy followed by a boost, whereas T1 and T2 prostate tumors had limited treatment volumes, with maximal margins of 2 cm.

### Secondary endpoints

Secondary outcome criteria were quality of life (QOL) [8], psychosocial status using Hospital Anxiety-Depression Scale (HAD) [25], and also quantitative measurement of C-reactive protein (CRP), as an indicator of systemic inflammation. A self-rating questioner was applied for evaluating the impact of symptoms on daily life activities, including a scale from 0 (no effect on daily activity) to 4 (Afraid to leave home with significant restriction in social life). The patients filled out HAD form weekly based on their feelings during the past week. The total scores 0–7 was considered normal, 8–10 borderline and higher than 11 was regarded as abnormal (depression or anxiety).

As part of the protocol, for measurement of CRP, serum samples were obtained at baseline, week 2, and at the subsequent week 6 visits. Quantitative testing for CRP was performed by using CRP-Latex Bionik slide agglutination test kit with a specificity of 95.6% and specificity of 96.2%. immunoturbidimetric assay. We appraised the psychosocial status of patients by applying HAD Scale. Patients were requested to fill out the HAD form weekly, considering their feelings during the past week.

### Sample size, study and statistical analysis

The sample size was estimated as 18 per group based on earlier experience and the pre- and post-intervention standard deviations of 1 in order to reach a mean difference (reduction in total clinical score) of 1 with the following specifications and using the sample size eq. (*N* = (z1 − α2/−z1 − β) 2 (SD1+ SD2) 2/d2) for comparing two means; the estimated sample size was increased to 19 per group to take account of potential attrition 10% (α = 0.01; β = 0.1). We used the Shapiro-Wilk test to test whether data were normally distributed. Descriptive baseline characteristics for two groups (Aloe ointment and placebo ointment) comparisons were tabulated as mean ± SD, median (inter-quartile range) or as percentages. Comparing between two groups for categorical data were statistically analyzed using chi- square or Fisher-exact test and for continuous data were statistically analyzed using t-test and Mann-Whitney U test. The primary efficacy was examined using intention-to-treat analysis. We used a generalized estimating equation (GEE) model to estimate the differences in values of endpoints at each time point between the two groups (between group effects). Within group effects were assessed with the Friedman test. A *p* value of 0.05 or less was considered statistically significant. Data were analyzed using IBM SPSS statistics version 16 and stata version 10.

### Ethics

The ethical committee of Mazandaran University of Medical Sciences approved the study protocol (Ethics Code; IR.MAZUMS.REC.94–1196). All patients received verbal and printed information, and all provided written consent before entry into this study.

## Results

### Participants

**Fig. 1:**
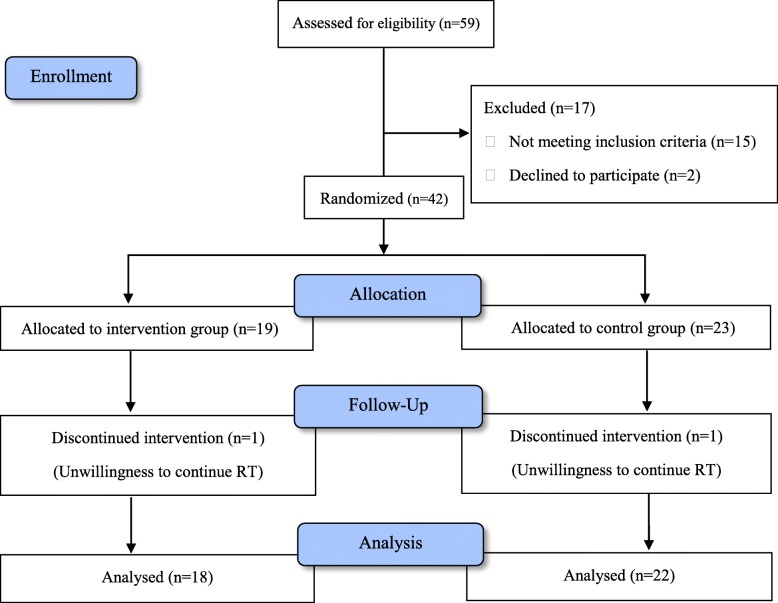
Flowchart of the study

Over a period of 13 months, 42 patients completed a cycle of external-beam radiation therapy to the pelvis. They were randomly assigned to receive Aloe ointment (*n* = 19) or placebo ointment (*n* = 23). Demographic and baseline clinical characteristics of enrolled patients were presented in Table 1. All patients were followed-up regularly. However, two patients (1 on each arm) withdrew the study, because of elective discontinuation of RT. Average patient age was 62.5 years, with no significant difference between groups. The rectal ointments were well tolerated in all patients. No patient in either group experienced any adverse effects. Our patients were objectively monitored with a patient diary. Compliance was good as assessed by comparing the actual and estimated volumes of residual ointment. The groups were similar with respect to gender ratio, body mass index, dose of radiotherapy in each session, concomitant medication and comorbidities (Table 1). Flow diagram of the study population selection is displayed in Fig. 1.

**Table 1:** Demographic and clinical features of patients

Characteristics	Aloe (*n* = 19)	Placebo (*n* = 23)
Age, mean ± SD (absolute range), y	61.5 ± 13.1	63.3 ± 11.8
Male sex, No. (%)	11 (57.9%)	16 (69.6%)
BMI, mean (SD), kg/m^2^	25.8 ± 3.2	25.3 ± 2.8
Dose of radiotherapy in each session, cGy	181.5 ± 4.8	186.3 ± 9.3
Total dose of radiotherapy received, cGy	5944.4 ± 1157.9	5721.7 ± 1111.2
**Concomitant medication, No(%)**		
NSAIDs	1 (5.3)	1 (4.3)
Opioid	2 (10.5)	1 (4.3)
Anticholinergic	0 (0)	2 (8.7)
Alpha Blocker	2 (10.5)	2 (8.7)
Cardiovascular	10 (52.6)	8 (34.8)
**Condition, No(%)**		
HTN	7 (36.8)	6 (26.1)
Diabetes	4 (21.05)	8 (34.8)
Dyslipidemia	2 (10.5)	4 (17.4)
IHD	2 (10.5)	2 (8.7)
Pelvic Surgery	4 (21.05)	7 (30.4)

*BMI* body–mass index, *HTN* hypertension, *NSAIDs* non-steroidal, anti-inflammatory drugs, *SD* standard deviation

### Effect of *Aloe vera* rectal ointment on symptoms

**Fig. 2:**
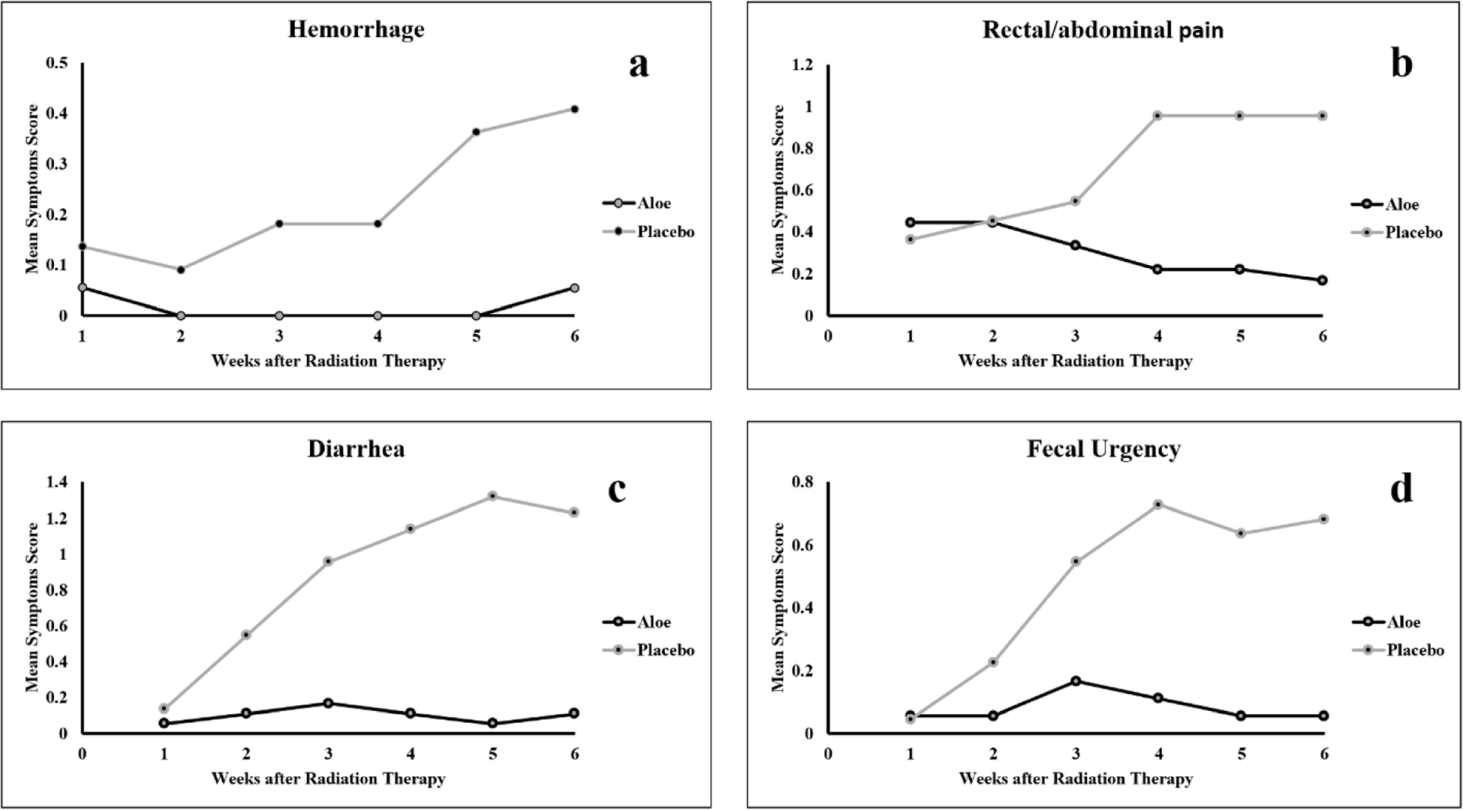
Clinical Symptoms trend of changes during six weeks of follow up: hemorrhage trend of changes (**a**), abdominal/rectal pain trend of changes (**b**), diarrhea trend of changes (**c**), Fecal urgency trend of changes (**d**)

Overall 65.2% of patients receiving placebo developed some degree of proctitis as compared with only 5% of patients receiving Aloe. The incidence of patient-derived toxicity factors is shown in Table 2. With exception of rectal pain, cystitis, and constipation, all measures of clinical presentation toxicity were significantly better with prevention with Aloe and (Table 2). Figure 2 shows outcomes by applying Aloe or placebo ointments over 6 weeks of the study.

**Table 2:** Trends of Changes in Clinical Presentation in *Aloe vera* and Placebo During a Six-Week Follow-up

		Week		Effect	
		1 Median (Q1,Q3)^a^	6 Median (Q1,Q3)^a^	Within group	Between group
**Rectal Bleeding**	***Aloe vera***	< 0.001 (< 0.001- < 0.001)	< 0.001 (< 0.001- < 0.001)	0.55	**< 0.001**
	**Placebo**	< 0.001 (< 0.001- < 0.001)	< 0.001 (< 0.001–1)	0.04	
**Rectal Pain**	**Aloe vera**	< 0.001 (< 0.001–1)	< 0.001 (< 0.001- < 0.001)	0.15	**0.14**
	**Placebo**	< 0.001 (< 0.001–1)	1 (< 0.001–2)	< 0.001	
**Diarrhea**	**Aloe vera**	< 0.001 (< 0.001- < 0.001)	< 0.001 (< 0.001- < 0.001)	0.54	**< 0.001**
	**Placebo**	< 0.001 (< 0.001- < 0.001)	1 (1–1.25)	< 0.001	
**Fecal urgency**	**Aloe vera**	< 0.001 (< 0.001- < 0.001)	< 0.001 (< 0.001- < 0.001)	0.42	**0.001**
	**Placebo**	< 0.001 (< 0.001- < 0.001)	1 (< 0.001–1)	< 0.001	
**Total Clinical Symptoms**	**Aloe vera**	< 0.001 (< 0.001–1)	< 0.001 (< 0.001–0.75)	0.13	**< 0.001**
	**Placebo**	< 0.001 (< 0.001–1)	2.5 (1.75–4.25)	< 0.001	
**Proctitis**	**Aloe vera**	< 0.001 (< 0.001- < 0.001)	< 0.001 (< 0.001- < 0.001)	0.7	**0.92**
	**Placebo**	< 0.001 (< 0.001- < 0.001)	< 0.001 (< 0.001- < 0.001)	0.65	
**Cystitis**	**Aloe vera**	1 (< 0.001–1)	0.5 (< 0.001–1.75)	0.7	**0.91**
	**Placebo**	< 0.001 (< 0.001–1)	1 (< 0.001–2)	0.06	
**RTOG Total**	**Aloe vera**	1 (< 0.001–1)	0.5 (< 0.001–1.75)	0.8	**0.03**
	**Placebo**	1 (< 0.001–2)	2 (1–3)	< 0.001	
**QOL**	**Aloe vera**	< 0.001 (< 0.001- < 0.001)	< 0.001 (< 0.001- < 0.001)	0.74	**< 0.001**
	**Placebo**	< 0.001 (< 0.001- < 0.001)	1 (< 0.001–1)	0.001	
**CRP**	**Aloe vera**	5.1 (2.1–11)	2.5 (2.0–4.5)	0.02	**0.009**
	**Placebo**	4.9 (2.0–6.3)	7.1 (4.27–17.2)	0.001	

*RTOG* radiation therapy oncology group, *QOL* Quality of life, *CRP* C-Reactive Protein

^a^ median [Percentile 25th - 75th]

As displayed in Table 2, the preventive effect of *Aloe vera*, in comparison to the placebo, is most significant for diarrhea, rectal bleeding and fecal urgency. As illustrated in Fig. 2, the average scores in placebo arm followed an upward trend during 6 weeks of study, while it remained almost unchanged in Aloe group.

### Secondary end points

Quality of life (QOL), depression and anxiety for assessment of psychosocial status of patients, and quantitative CRP were evaluated as secondary endpoints. The median lifestyle score was significantly improved with *Aloe vera*, whereas in placebo group, the overall score value was significantly increased, indicating the worsening quality of life during the study period. Improvement in the depression status in HAD scale in Aloe group was also observed (*p* = 0.049). However, anxiety scores in psychosocial status of patients were not statistically different (*p* = 0.053), but still favored Aloe group. Quantitative measurement of C-reactive protein (CRP) showed significant improvement in intervention group (*p* = 0.009).

## Discussion

Proctitis is an inflammatory process of rectal mucosa and the most common side effect of RT for malignant pelvic disease [1, 2]. The gastrointestinal tract is susceptible to pelvic RT, because of its rapid dividing mucosa [26]. The lower colon and the rectum are particularly involved as the most common site of injury, because of its close position to the radiation exposure region. Shielding the rectum during radiation is inapplicable or difficult [27]. Therefore, the risk of radiation-induced damage to the rectum is feasible and patients suffer from ARP symptoms. Alterations in bowel habits and diarrhea, which are the most frequent distress experienced by the patients, are of special importance [28].

Attempts to use adjunctive medical therapy (eg, amifostine) [29, 30] along with modification in radiation therapy techniques [31, 32] have been applied to prevent the development of radiation proctitis, but unfortunately, they have had only a minimal effect and are not widely used. Therefore, an effective and safe method for preventing or minimizing radiation-induced proctitis remains unanswered.

Results of this study indicate the topical application of *Aloe vera* reduced the incidence of ARP in patients undergoing radiotherapy for malignant pelvic disease, targeting the possible pathophysiology of injury. To our knowledge, this is the first double-blind placebo-controlled, clinical trial of *Aloe vera* in prevention of ARP. The results of the present study were so inspiring, revealing that prevention with *Aloe vera* 3% ointment at a dose of 1 g/day, 1 day before and during radiotherapy for 6 weeks, is significantly more effective compared to the placebo group. In this study, *Aloe vera* significantly decreased the occurrence of all measured clinical presentations and RTOG toxicity except rectal pain, cystitis, and constipation. Fewer incidences of adverse effects of RT itself contributes to further improvement in the quality of life of the patients, as the patients in Aloe group experienced an enhanced quality of life, and psychological state with fewer scores of depression and anxiety. In the present study, changes of quantitative CRP were monitored for evaluation of systemic inflammation. This biomarker reflects total systemic load of inflammation in various diseases and potentiate production of pro-inflammatory cytokines. As stated in Table 2, the burden of inflammation followed a decreasing trend after initiation of Aloe ointment, suggesting an objective indicator of improvement in ARP.

Acute radiation cystitis presents most commonly as dysuria, increased urinary frequency and urgency. These clinical features are nonspecific, and can also be caused by bladder infection or malignancy [33]. This might explain why cystitis had an insignificant effect in our study. Since more than 57% of our patients had prostate or bladder cancer and the symptoms of cystitis were experienced by almost all of them.

As mentioned before, the direct effect of the radiation or the subsequent ischemia induced by the radiation may form reactive oxygen species. These noxious agents have the main role in pathophysiology of ARP. Experimental evidence as well as clinical studies support the use of antioxidants in radiation proctitis [8, 34]. *Aloe vera* is considered a non-toxic agent with no known side-effects, and is widely available in the world. It carries out its effectiveness through scavenging reactive oxygen metabolites. Besides, one of the active component present in Aloe gel has anti-inflammatory activity via an inhibitory action on the arachidonic acid pathway through cyclooxygenase and reduction of prostaglandin E2 as well as increased infiltration of leucocytes [35]. Previous studies have shown that topical *Aloe vera* is beneficial in reducing radiation-induced mucositis and skin side effects, including erythema, itching, and pain [13, 36, 37]. Therefore, *Aloe vera*, with its anti-inflammatory, anti-oxidant effects, and wound healing properties, enhances healing of mucosal lesions and reduces related symptoms and might be effective in the underlying pathology of the damage to irradiated rectal tissue.

One limitation of our study is that we did not apply the rectosigmoidoscopic assessment. However, according to previous reports, such mechanical intervention during the acute phase should be avoided to prevent worsening of the condition. Besides, it has the risk of irreversible damage to the internal sphincter and the resultant incontinence and infection [38]. Therefore, we did not find sigmoidoscopy ethical for the first clinical trial of *Aloe vera* at this point in time. In addition, the findings of the present study should be confirmed in a larger population of patients undergoing pelvic RT.

## Conclusion

*Aloe vera* ointment was superior to placebo in prevention of ARP in patients during RT. It prevented the symptoms of radiation- induced proctitis, especially diarrhea, hemorrhage, and fecal urgency. *Aloe vera* also decrease the burden of systemic inflammation and enhanced quality of life of patients without causing significant adverse event. The results of this study is so promising, suggesting the use of this natural product for prevention of ARP in patients undergoing RT.

## Data Availability

• The datasets used and/or analysed during the current study available from the corresponding author on reasonable request. The raw SPSS file of this study before analysis is available upon your request.

## Abbreviations

ARP: Acute radiation-induced proctitis
RT: Radiotherapy
QOL: Quality of life
HAD: Psychosocial status using Hospital Anxiety-Depression Scale
CRP: C - reactive protein
BMI: Body–Mass Index
HTN: Hypertension
NSAIDs: Non-steroidal, anti-inflammatory drugs
